# Epidemiological and Virological Characteristics of H9N2 Avian Influenza Virus in Jiangsu Province, China, 2024

**DOI:** 10.3390/v18060687

**Published:** 2026-06-20

**Authors:** Xue Gao, Huiyan Yu, Na Zhang, Liqi Liu, Jing Tong, Xian Qi, Haodi Huang, Shenjiao Wang, Zi Li, Yangguang Du, Liguo Zhu

**Affiliations:** 1 Department of Microbiology, Xuzhou Center for Disease Control and Prevention, Xuzhou 221006, China; gaoxue8026@163.com (X.G.); tongjing80@163.com (J.T.); 2 Department of Acute Infectious Disease Control and Prevention, Jiangsu Provincial Center for Disease Control and Prevention, Nanjing 210008, China; yuhy86@126.com (H.Y.); qixiansyc@163.com (X.Q.); huanghd@jscdc.com (H.H.); wangsj@jscdc.com (S.W.); 3 National Institute for Viral Disease Control and Prevention, Chinese Center for Disease Control and Prevention, Beijing 102206, China; znning21@126.com (N.Z.); liulq@ivdc.chinacdc.cn (L.L.)

**Keywords:** H9N2 avian influenza virus, genetic evolution, receptor binding properties, seroepidemiology, pandemic risk

## Abstract

H9N2 avian influenza viruses inherently carry cross-species transmission potential, making continuous surveillance critical for pandemic prevention. This study focused on monitoring the 2024 H9N2 epidemic in Jiangsu Province’s external environment, analyzing its molecular evolution and receptor binding properties, assessing cross-species transmission and pandemic risks, and investigating serological antibody levels across different human populations. Environmental samples were collected from live poultry markets, farms, slaughterhouses, and bird habitats across Jiangsu, screened via quantitative PCR (qPCR), with positive samples used for virus isolation and whole-genome sequencing. Receptor binding properties were tested by hemagglutination assay, and H9N2 antibody levels were measured in 370 occupationally exposed individuals and 240 non-exposed individuals using hemagglutination inhibition (HI) assays. Among the 5779 collected samples, 6.89% tested H9N2-positive, and 12 strains belonging to the Eurasian lineage Y280-like clade G57 genotype were successfully isolated. All strains carried the HA-Q226L mutation, with 11 showing preferential binding to human α-2,6 receptors and one strain possessing dual receptor binding capability. Internal genes harbored mammalian adaptation mutations, and M2 proteins contained mutations conferring complete resistance to amantadine-class antiviral drugs. Serological tests revealed antibody positive rates of 4.05% in exposed populations and 2.5% in non-exposed populations, with no statistically significant difference between groups. These findings confirm that Jiangsu’s circulating H9N2 viruses have acquired human receptor preference and mammalian adaptation, posing silent infection and pandemic risks. Enhanced surveillance and the development of candidate vaccine stockpiles are strongly recommended.

## 1. Introduction

Avian influenza virus (AIV) is a highly infectious pathogen characterized by high genomic variability and cross-host transmission capability, continuously threatening the global poultry industry and public health safety [1]. Among various subtypes, H9N2 AIV has evolved from a mere low-pathogenicity threat into a subtype that is widely distributed among poultry worldwide and exerts significant evolutionary influence.

Since 2016, H9N2 has gradually replaced H5N6 and H7N9 as the predominant AIV subtype in Chinese chicken and duck populations [2]. Although H9N2 infection in poultry manifests as mild clinical symptoms, it can provide backbone genes to H5N6, H7N9, H10N8, promoting the emergence of novel reassortant viruses (such as H3N8) and posing a potential public health threat [3,4,5,6].

In recent years, the proportion of environmental H9N2 strains carrying human receptor binding site mutations (HA-Q226L) has significantly increased, with human infection cases rising annually. This subtype virus has gradually breached species barriers and has become one of the influenza viruses with the greatest potential for a pandemic [7].

Live poultry markets, as places with high concentration of poultry and their secretions, are commonly considered primary sites for virus preservation, transmission, and mutation [8,9]. Epidemiological evidence indicates that the majority of H9N2 infection cases have a clear exposure history to live poultry markets or contact with poultry [10]. However, current surveillance largely focuses on environmental pathology or clinical case reports, overlooking potential silent infections and serological antibody dynamics among exposed populations [11,12].

This study aims to reveal the potential pathways of H9N2 virus transmission from the environment to humans through virological surveillance of the external environment in live poultry markets and serological surveys of key populations in Jiangsu Province in 2024, and to assess the risk of its evolution into a human pandemic strain from a One Health perspective.

## 2. Materials and Methods

### 2.1. Sample Sources

A total of 5779 environmental samples were collected in 2024 from live poultry markets, farms, slaughterhouses, and bird habitats in Jiangsu Province (including fecal samples, poultry drinking water, poultry washing wastewater, and cage surface swabs). Samples were stored at −80 °C after collection. Serum samples were collected from occupationally exposed populations (poultry industry workers) at 5 monitoring sites in Jiangsu Province (*n* = 370) and non-occupationally exposed populations (*n* = 240). All serum samples were stored at −20 °C after collection.

### 2.2. Quantitative Real-Time PCR Nucleic Acid Identification

The 200 μL samples were extracted according to the nucleic acid extraction kit instructions (Cat. No.: T332, Xi’an Tianlong Technology, Xi’an, China). The obtained RNA was then amplified using the Fast Virus 1-Step Master Mix (Thermo Fisher, Vilnius, Lithuania) with H9 and N2 primers and probes. (Shanghai Sangon Bioengineering, Shanghai, China). H9HA: (F: 5′-CAAGCTGGAATCTGARGGAACTTACA-3′; R: 5′-GCATCTGCAAGATCCATTGGACAT-3′; P: 5′-CCCAGAACARGAAGGCAGCAAACCCCATTG-3′). H9NA: (F: 5′-AGCCGAGTATAAAAATTGGTCAAA-3′, R: 5′-CACCTGCAGAAAGCCTAATTGAGE-3′; P: 5′-TGTCTAATTACAGGGTTCGCTCCTTTCTC-3′).

### 2.3. Virus Isolation and Culture

A total of 398 qPCR-positive samples were each inoculated with 200 μL into 9-day-old specific pathogen-free (SPF) chicken embryos, cultured at 37 °C for 48 h, then overnight at 4 °C before harvesting allantoic fluid. Hemagglutination assays were performed using 1% turkey red blood cells (provided by the National Influenza Center) to determine virus HA titers. Harvested strains were stored at −80 °C.

### 2.4. Viral Genome Sequencing and Genetic Evolution Analysis

RNA extracted by qPCR was subjected to whole-genome specific amplification using the SuperScript^®^ III One-Step RT-PCR System kit (Invitrogen, Waltham, MA, USA); PCR products were purified using the QIAquick PCR Purification kit (Qiagen, Hilden, Germany); and DNA sequencing libraries were constructed using the Nextera XT DNA Sample Preparation kit (Illumina, San Diego, CA, USA). Whole-genome deep sequencing was performed using Illumina platform’s Miniseq sequencer.

Obtained isolate sequences were subjected to BLAST analysis in the NCBI influenza database (https://www.ncbi.nlm.nih.gov/genomes/FLU/Database/nph-select.cgi, accessed on 16 June 2026), and representative strains and vaccine strains from various H9N2 subtype AIV lineages were downloaded as references, including classical H9N2 vaccine strains, A/chicken/Shanghai/F/98 (H9N2), A/chicken/Guangdong/SS/94 (H9N2), A/chicken/Shandong/6/96 (H9N2), and A/chicken/Guangxi/10/99 (H9N2) and WHO candidate vaccine strains: A/HongKong/1073/99 (H9N2), A/chicken/HongKong/G9/97 (H9N2), A/HongKong/33982/2009 (H9N2), A/Hong Kong/308/2014 (H9N2), A/Anhui/Lujiang/39/2018 (H9N2), A/Oman/2747/2019 (H9N2) and A/Anhui-Tianjiaan/11086/2022 (H9N2) [13]. Multiple sequence alignment was performed using MEGA 12 software, phylogenetic trees were constructed using the neighbor-joining method (NJ), and branch support rates were calculated by performing 1000 bootstrap replicates to assess phylogenetic tree reliability.

### 2.5. Hemagglutination Assays

A volume of 50 µL PBS was added to each well in columns 2–12 of 96-well hemagglutination plates, and 100 µL test virus was added to each well in column 1. After mixing each well in column 1, 50 µL virus suspension was drawn and serial two-fold dilutions were made from column 1 to column 11. After mixing column 11, the 50 µL virus suspension was discarded. A 50 μL red blood cell suspension was added to each well of the microplate, with column 12 serving as red blood cell control. The microplate was gently tapped to thoroughly mix red blood cells with virus. Turkey and chicken red blood cells were observed after 30 min of static incubation at room temperature, while guinea pig, horse, sheep, and rabbit red blood cells were observed after 45 min. Detailed detection methods are described in the National Influenza Surveillance Technical Guidelines (2017 Edition), https://ivdc.chinacdc.cn/cnic/fascc/201802/t20180202_158592.htm (accessed on 16 June 2026).

### 2.6. Receptor Binding Assays

Biotinylated α-2,3 and α-2,6 sialic acid glycans (Glytech, Hammond, LA, USA) were used as receptor analogs. PBS was used to prepare serial dilutions (0.16 μg/mL, 0.32 μg/mL, 0.63 μg/mL, 1.25 μg/mL, 2.5 μg/mL, 5 μg/mL, 10 μg/mL) of both receptor analogs in 96-well plates, incubated overnight at 4 °C; after washing with cold PBS containing 0.1% Tween 20 (PBST), virus at HA = 64 was added and incubated at 4 °C for 12 h; after washing, influenza hemagglutinin broad-spectrum monoclonal antibody (Suben Yuanhe, Suzhou, China) was added and incubated at 4 °C for 2 h; after washing, HRP-labeled goat anti-human antibody (Yiqiao Shenzhou, Beijing, China) was added and incubated at 4 °C for 2 h; after washing, TMB dual-component chromogenic solution was added, incubated at room temperature for 10 min, then stopped with 0.5 mol/L sulfuric acid, and absorbance was measured at 450 nm wavelength using an enzyme-linked immunosorbent assay reader.

### 2.7. Hemagglutination Inhibition Assays

The virus was adjusted to 8 hemagglutination units (HAU)/50 μL according to influenza virus hemagglutination titer units. A total of 100 μL serum was mixed with 300 μL receptor-destroying enzyme (Denka Seiken, Tokyo, Japan), incubated overnight in a 37 °C water bath, then 30 min at 56 °C water bath, followed by addition of 600 μL PBS and mixing. Additionally, 25 μL of initially diluted serum was taken and serially diluted two-fold with PBS; then 25 μL virus solution was added to each dilution, incubated at room temperature for 20 min, followed by addition of 50 μL 1% turkey red blood cells, and results were observed after 30 min of static incubation. An antibody inhibition titer of ≥1:80 was used as the criterion for seropositivity. The 1% turkey red blood cells and standard sera were provided by the Chinese National Influenza Center.

### 2.8. Statistical Analysis

Statistical analysis was performed using SPSS 26.0 software. Comparison of antibody positive rates between occupationally exposed and non-occupationally exposed populations was performed using a two-sided Fisher’s exact test. *p* < 0.05 was considered statistically significant.

## 3. Results

### 3.1. Environmental Sample Information Collected in Jiangsu Province in 2024

A total of 5779 external environment samples were collected in Jiangsu Province in 2024, including 3113 from live poultry markets, 1802 from farms, 131 from slaughterhouses, and 733 from bird habitats. The qPCR method identified 398 (6.89%) H9N2 subtype positive avian influenza viruses. Live poultry markets had the highest number of positive detections, while slaughterhouses had the highest positive detection rate (Table 1).

### 3.2. Isolated Strain Information

Twelve H9N2 subtype AIV isolates were obtained from eight monitoring sites including Hai’an City, Zhenjiang New Area, and Changzhou Economic Development Zone, with 11 strains from urban and rural live poultry markets and one strain (YC0277) from a poultry slaughterhouse (Table 2).

### 3.3. Whole-Genome Evolution Analysis

#### 3.3.1. Phylogenetic and Genetic Evolution Analysis of HA and NA

Genomic characterization showed that the 12 H9N2 isolates obtained in this study exhibited high genetic stability, with HA and NA gene nucleotide identities of 95.87–100% and 91.01–100%, respectively. Compared to recently circulating avian reference strains (from Fujian, Hunan, Chongqing, Vietnam, and other regions), the HA and NA gene identities were maintained at 97.53–98.28% and 96.84–97.87%, respectively, suggesting close phylogenetic relationships between the isolated strains and currently circulating strains in East Asia and Southeast Asia.

**Figure 1:**
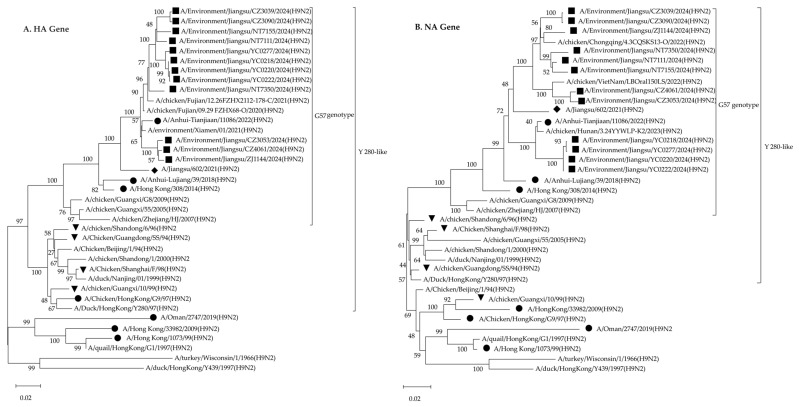
Phylogenetic tree of surface genes of H9N2 AIV isolates in Jiangsu Province, 2024. (**A**) HA gene phylogenetic tree. (**B**) NA gene phylogenetic tree. ■: H9N2 subtype strains in this study; ▼: H9N2 classical vaccine strains; ●: WHO candidate vaccine strains; ◆: human avian influenza strains from Jiangsu Province.

Phylogenetic analysis (Figure 1) showed that the HA and NA genes of all 12 isolates belonged to the Y280-like branch of the Eurasian lineage and were further identified as the currently predominant G57 genotype in China. Notably, these environmental isolates clustered in the same sub-branch as the human infection case strain A/Jiangsu/602/2021 (H9N2) reported in Jiangsu Province in 2021, showing obvious “humanized” clustering characteristics. Additionally, the 12 isolates were within the same major branch as World Health Organization (WHO)-recommended candidate vaccine strains A/Anhui-Tianjiaan/11086/2022 (H9N2) and A/Hong Kong/308/2014 (H9N2), demonstrating good genetic compatibility; they were significantly distantly related to early classical vaccine strains (such as A/chicken/Guangdong/SS/94), indicating that H9N2 viruses have undergone dramatic antigenic drift during long-term circulation.

#### 3.3.2. Phylogenetic and Genetic Evolution Analysis of Internal Genes

**Figure 2:**
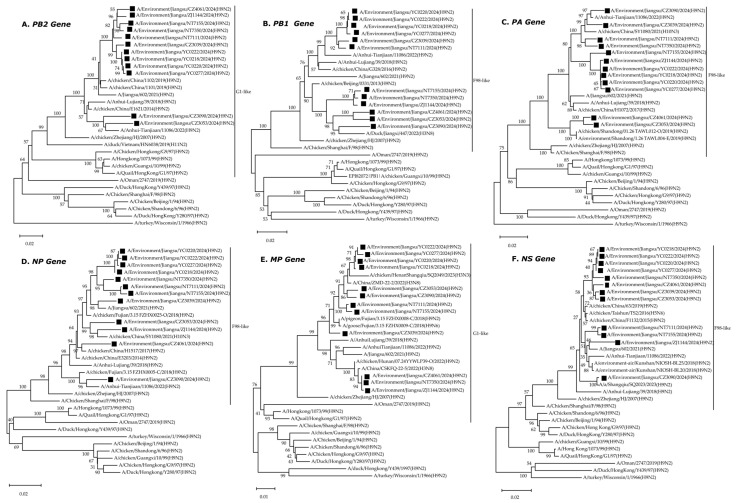
Phylogenetic analysis of the internal genes of H9N2 subtype avian influenza viruses isolated from Jiangsu in 2024 using the neighbor-joining method. (**A**) PB2 gene phylogenetic tree. (**B**) PB1 gene phylogenetic tree. (**C**) PA gene phylogenetic tree. (**D**) NP gene phylogenetic tree. (**E**) MP gene phylogenetic tree. (**F**) NS gene phylogenetic tree. ■: H9N2 subtype strains in this study.

The six internal genes (PB2, PB1, PA, NP, M, NS) of the 12 H9N2 isolates showed high genetic conservation and epidemic stability, with nucleotide sequence identity as follows: PB2: 92.39–100%, PB1: 92.95–100%, PA: 92.88–100%, NP: 93.80–100%, M: 95.03–100%, NS: 96.18–100%. Phylogenetic analysis revealed complex gene reassortment characteristics, with PB1, PA, NP, and NS genes clustering in the F98-like branch (Figure 2B–D,F); PB2and MP genes showed different evolutionary origins, both belonging to the G1-like branch (Figure 2A,E). This gene combination pattern (PB1 + PA + NP + NS from F98-like, PB2 + M from G1-like) indicates that all 12 Jiangsu environmental isolates belong to the predominant G57 genotype found in Chinese poultry in recent years.

### 3.4. Molecular Evolution Characteristics

#### 3.4.1. HA Protein

Analysis of pathogenicity motifs and receptor binding preference molecular characteristics showed that all 12 H9N2 isolates had HA protein cleavage site motifs of PSRSSR↓GLF, consistent with typical low pathogenic avian influenza virus characteristics. Compared to early vaccine strains, the second amino acid at this site evolved from Ala→Ser and remained highly consistent with current candidate vaccine strains. Regarding critical receptor binding sites, all isolates carried the key mutations H183N, A190T/V, and Q226L (encoded by H3).

Analysis of glycosylation sites revealed that, except for ZJ1144 and CZ3053 strains, the remaining 10 isolates possessed five conserved N-linked glycosylation sites in the HA1 subunit: 29NST,141NVS, 298NTT, 305NVS, and 313NCS. The predicted 82NPS site was excluded because proline at the second position of the N-X-S/T motif sterically inhibits glycosylation, which does not conform to the canonical N-glycosylation definition. Compared to early vaccine strains, the 10 isolates showed obvious glycosylation shifts: loss of the 218NRT site due to T220I mutation, accompanied by gain of the 313NCS site due to P315S mutation. The CZ3053 strain also contained five glycosylation sites in HA1, but its 305 position was NLS (others were NVS); the ZJ1144 strain retained the 218NRT site due to lack of T220I mutation and had a total of six conserved glycosylation sites in HA1. All isolates contained two glycosylation sites in the HA2 subunit (492NGT, 551NGS), reflecting structural stability in maintaining membrane fusion function (Table 3).

#### 3.4.2. NA Protein

All 12 isolates showed characteristic deletion of 63–65 amino acids in the NA protein stalk region. Regarding drug sensitivity, no known neuraminidase inhibitor resistance mutations such as E119D, D151E, E276D, R292K, N294S, and R371K were detected. Analysis of auxiliary sialic acid binding sites in the NA protein found that the 431–433 site was highly conserved, being PQE in all isolates; the 368–370 site showed some geographical correlation: except for the four strains isolated from Yancheng City, the remaining eight were the NSS motif; the 399–401 site presented three forms: DSD, DSV, and DGD. Glycosylation site analysis revealed that all 12 isolates harbored potential N-glycosylation sites at NA protein positions 69 (NST), 146 (NGT), 200 (NAT), and 234 (NGT). At position 368, four strains including YC0218 and YC0220 carried the NDS motif, while the remaining eight strains possessed the NSS motif. Additionally, nine strains contained a potential glycosylation site at position 86 (NWS). Notably, although six isolates contained the NPS motif at position 44, this sequence does not conform to the canonical N-X-S/T consensus (where X ≠ proline) due to the presence of proline at the X position, and thus represents an invalid putative N-glycosylation site. In addition, one isolate had NMT at position 306 and partial isolates contained NWS at position 402 (Table 4).

#### 3.4.3. Molecular Characteristics of Internal Genes

Internal gene analysis revealed that all 12 isolates carried multiple key adaptive mutations in the polymerase complex (PB2, PB1, and PA). All isolate PB2 proteins contained L89V, L134H, M147I, I292V, R340K, R389K, and A588V mutations, with A588V being a key determinant for enhancing influenza virus polymerase activity in mammalian cells; isolates CZ4061, NT7155, NT7350, and ZJ1144 also had V613I mutation but lacked classical E627V and D701N mutations. Synergistic mutations in isolate PB1 (I368V, L473V, and D622G) and PA proteins (I70V, P224S, K356R, N383D, A515T, and 672L) further supported virus preference for mammalian host environments. NP protein also showed strong host adaptation characteristics, with all isolates having the key mutations V41I, V183I, D210E, V352M, K398Q, and L479F; except for a few strains like CZ4061 and YC0218, most (7/12) isolates also had I353V mutation. In M protein, all isolate M2 proteins had V27A and S31N mutations determining adamantane drug resistance; M1 proteins all had N30D and T215A mutations. NS protein contained specific mutations proven to regulate host interferon antagonism (P42S, D97E, P114S, M124V, and E172K), which may help increase the pathogenicity of H9N2 circulating strains.

#### 3.4.4. Hemagglutination Assay Results of H9N2 Subtype Avian Influenza Virus with Different Animal Red Blood Cells

All 12 isolates effectively agglutinated chicken, turkey, and guinea pig red blood cells, with most strains showing highly consistent hemagglutination (HA) titers among the three red blood cell types (difference ≤4-fold), suggesting robust recognition capacity for both avian and mammalian receptors. However, significant differences existed in binding specificity among different strains. The CZ3039, CZ3090, and NT7350 strains showed obvious receptor binding preference changes: chicken red blood cell HA titers were significantly lower than turkey and guinea pig red blood cells (difference ≥8-fold). Additionally, except for the CZ3053 strain which retained agglutination capacity with horse, rabbit, and sheep red blood cells, the remaining 11 strains lost agglutination activity against these three types of red blood cells (Table 5).

### 3.5. Results of Receptor Binding Assays

**Figure 3:**
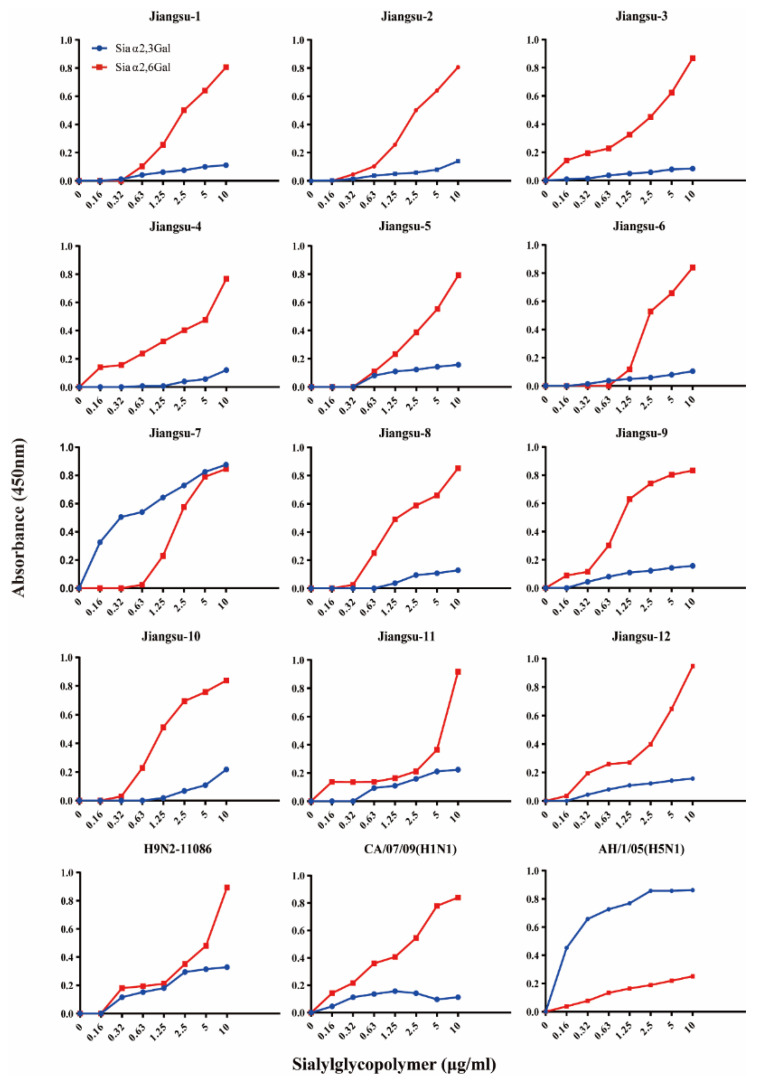
Receptor binding properties of 12 H9N2 AIV isolates.

Unlike traditional avian influenza viruses that preferentially bind α-2,3 sialic acid receptors, the H9N2 subtype avian influenza viruses isolated in this study all demonstrated binding advantage to human α-2,6 sialic acid receptors. Eleven virus strains especially showed preferential binding patterns to α-2,6 sialic acid receptors under experimental conditions, with relatively weak binding signals to α-2,3 sialic acid receptors. Only strain CZ3053 simultaneously showed affinity for both types of sialic acid receptors (Figure 3).

### 3.6. Serological Detection Results

**Figure 4:**
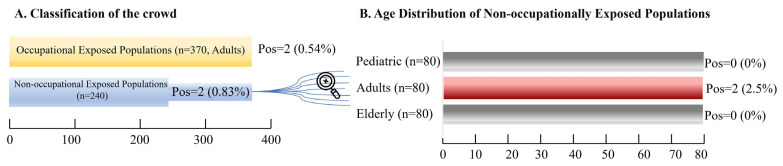
Serological detection of H9N2 antibodies among different populations. The magnifying glass branching symbol separates data of occupationally exposed and non-occupationally exposed populations. Grey bars represent pediatric and elderly subgroups, while the red bar represents the adult subgroup of non-occupationally exposed individuals. Pos = number of seropositive samples, percentage in parentheses indicates seropositive rate of each subgroup.

Serological surveillance results showed that the H9N2 influenza virus presents low-level sporadic infection in human populations. The antibody positivity rate in occupationally exposed populations was 4.05% (15/370), while the positivity rate in non-exposed populations was 2.5% (6/240). Chi-square test results indicated no statistically significant difference in serum antibody positivity rates between the two groups (*p* = 0.304), suggesting that among populations from whom serum was collected in Jiangsu Province in 2024, the H9N2 avian influenza virus poses similar infection risks across populations with different exposure levels. Further age stratification of the non-occupationally exposed population (*n* = 240) revealed that all six positive subjects were from the adult group (18–60 years old), with no positives detected in the pediatric group (0–18 years old) or elderly group (>60 years old) (Figure 4).

## 4. Discussion

This study conducted a systematic genomic, receptor binding, and serological assessment of 12 H9N2 viruses isolated from environmental samples in live poultry markets in Jiangsu Province in 2024. Results indicate that currently circulating H9N2 strains have shown trends toward humanization, receptor binding characteristics, and mammalian adaptation mutations, with low-level silent infection existing in human populations, posing severe challenges to current public health safety.

All 12 isolates in this study originated from live poultry market environments, indicating that live poultry markets remain key venues for H9N2 subtype AIV transmission, posing potential threats to human health. Phylogenetic analysis revealed that all 12 isolates belonged to genotype G57 in the Y280-like branch. This genotype has replaced G1 subtype as the absolute predominant strain in Chinese poultry since 2013 due to its powerful reassortment capability and environmental adaptability [14]. The high homology between environmental isolates in this study and the human strain A/Jiangsu/602/2021 (H9N2) from this region in 2021 suggests that this genotype virus has been continuously circulating in this region with established stable cross-species transmission potential. The high consistency between environmental and human strains also strongly demonstrates that live poultry market environments are important sources of human H9N2 infection, highlighting the importance of strengthening live poultry market surveillance [15].

Receptor binding characteristics are key for virus breakthrough of species barriers. Research found that 11/12 isolates lost agglutination ability against horse, sheep, and rabbit red blood cells (rich in α-2,3 receptors) and had high affinity for α-2,6 human receptors, consistent with reports from other high-incidence regions in China [16,17,18]. Q226L is a key marker for influenza virus adaptation to human respiratory tract, altering the spatial conformation of the receptor binding site to facilitate easier binding to human epithelial cells [19]. Additionally, compared to early vaccine strains, the loss of a glycosylation site at position 218 caused by T220I and the new glycosylation site at position 313 caused by P315S may constitute the virus’s glycan shield, achieving immune escape through spatial steric effects that mask key antigenic epitopes, thereby causing continuous silent infection in human populations [20].

Notably, dual receptor binding is a key determinant for cross-species transmission and pandemic potential in influenza viruses [21]. Strain CZ3053 not only possessed strong human receptor binding capacity but also retained agglutination activity against horse, sheep, and rabbit red blood cells. Analysis suggests that the HA-V306L mutation may be key for its acquisition of dual receptor binding capability. Amino acid substitution at position 306 may fine-tune the conformation of the receptor binding site through remote allosteric effects, enabling it to maintain avian α-2,3 receptor recognition capacity while further optimizing spatial adaptation to human α-2,6 receptors, thereby acquiring dual receptor binding characteristics.

All isolate PB2 proteins in this study carried a characteristic set of mutations (L89V, L134H, M147I, I292V, R340K, R389K, and A588V) [16]. Among these, M147I and R340K are key molecular markers of the G1-like lineage, closely related to enhanced polymerase activity and replication capacity in mammalian cells [22]. Additionally, combined with the commonly present mutations in PB1 protein (D622G) and PA protein (K356R, S409N, and A515T) related to virulence and polymerase activity, as well as adaptive mutations in NP protein that optimize nuclear transport efficiency (K398Q) and antagonize human restriction factor interference (V352M), these molecular characteristics functionally couple to provide a solid molecular basis for the H9N2 virus breaking through species barriers [23,24,25].

Serological results showed low H9N2 exposure levels in human populations in Jiangsu region in 2024, with no statistically significant difference between occupationally exposed (4.05%) and non-occupationally exposed (2.5%) groups. Based on our findings, H9N2 infection risk may not be limited to occupationally exposed populations, as general residents may also face infection risks through live poultry market environmental exposure or aerosol contact. Therefore, serological investigation and detection work should be strengthened for different populations. Meanwhile, both positive cases in the non-occupational group were from the adult group, possibly related to higher social activity frequency and environmental exposure opportunities in this age group. Since current antibody levels are far from sufficient to form population immune barriers, accelerating development and application of novel vaccines is a key measure for improving prevention and control effectiveness.

## 5. Conclusions

H9N2 viruses circulating in Jiangsu Province in 2024 have acquired human receptor preference, along with PB2-A588V and other internal gene compensatory adaptations, indicating pandemic potential. It is recommended to conduct continuous surveillance of the H9N2 subtype AIV while accelerating candidate vaccine strain matching and stockpiling work for current circulating branches to respond to potential public health crises.

## Figures and Tables

**Table 1:** Distribution and qPCR detection results of samples from different sources.

No.	Sample Source	Number of Samples Collected	Percentage of Samples Collected (%)	qPCR Positive Count	qPCR Positive Rate (%)
1	Live Poultry Market	3113	53.87	345	11.08
2	Breeding Farm	1802	31.18	16	0.89
3	Slaughterhouse	131	2.27	35	26.72
4	Bird Habitat	733	12.68	2	0.27
	Total	5779	100	398	

**Table 2:** Basic information of 12 H9N2 subtype AIV isolates.

Number	Strain	Abbreviation	Location	Type of Monitoring Site	Specimen Type
Jiangsu-1	A/Environment/Jiangsu/NT7155/2024(H9N2)	NT7155	Hai’an City	Urban and Rural Live Poultry Market	Wastewater from the cleaning of poultry
Jiangsu-2	A/Environment/Jiangsu/ZJ1144/2024(H9N2)	ZJ1144	Zhenjiang New District	Urban and Rural Live Poultry Markets	Poultry drinking water
Jiangsu-3	A/Environment/Jiangsu/CZ3090/ 2024(H9N2)	CZ3090	Economic Development Zone	Urban and Rural Live Poultry Market	Other
Jiangsu-4	A/Environment/Jiangsu/CZ3039/ 2024(H9N2)	CZ3039	Economic and Technological Development Zone	Urban and Rural Live Poultry Market	Surface swab from cage
Jiangsu-5	A/Environment/Jiangsu/NT7350/ 2024(H9N2)	NT7350	Chongchuan District	Urban and Rural Live Poultry Market	Poultry drinking water
Jiangsu-6	A/Environment/Jiangsu/CZ4061/ 2024(H9N2)	CZ4061	Xinbei District	Urban and Rural Live Poultry Market	Surface swab from cage
Jiangsu-7	A/Environment/Jiangsu/CZ3053/ 2024(H9N2)	CZ3053	Economic and Technological Development Zone	Urban and Rural Live Poultry Market	Other
Jiangsu-8	A/Environment/Jiangsu/NT7111/ 2024(H9N2)	NT7111	Hai’an City	Urban and Rural Live Poultry Market	Surface swab samples from cages
Jiangsu-9	A/Environment/Jiangsu/YC0220/ 2024(H9N2)	YC0220	Yandu District	Urban and Rural Live Poultry Market	Poultry drinking water
Jiangsu-10	A/Environment/Jiangsu/YC0222/ 2024(H9N2)	YC0222	Yandu District	Urban and Rural Live Poultry Market	Other
Jiangsu-11	A/Environment/Jiangsu/YC0277/ 2024(H9N2)	YC0277	Dongtai City	Poultry Slaughtering and Processing Plant	Swab sample from cutting board surface
Jiangsu-12	A/Environment/Jiangsu/YC0218/ 2024(H9N2)	YC0218	Yandu District	Urban and Rural Live Poultry Market	Poultry drinking water

**Table 3:** Analysis of cleavage sites, key amino acid residues, and potential glycosylation sites in the HA protein of isolates.

Strain	Cleavage Site	Receptor Binding Site (Encoded by H3)									Potential Glycosylation Sites							
	333–340	158	160	183	189	190	224	226	227	228	29	141	218	298	305	313	492	551
NT7155	PSRSSR↓GL	N	N	N	D	V	N	L	M	G	NST	NVS	-	NTT	NVS	NCS	NGT	NGS
ZJ1144	PSR SSR↓GL	N	N	N	D	V	H	L	M	G	NST	NVS	NRT	NTT	NVS	NCS	NGT	NGS
CZ3090	PSR SSR↓GL	N	N	N	D	V	N	L	M	G	NST	NVS	-	NTT	NVS	NCS	NGT	NGS
CZ3039	PSR SSR↓GL	N	N	N	D	V	N	L	M	G	NST	NVS	-	NTT	NVS	NCS	NGT	NGS
NT7350	PSR SSR↓GL	N	N	N	D	V	N	L	M	G	NST	NVS	-	NTT	NVS	NCS	NGT	NGS
CZ4061	PSRSSR↓GL	N	N	N	D	V	H	L	M	G	NST	NVS	-	NTT	NVS	NCS	NGT	NGS
CZ3053	PSR SSR↓GL	N	N	N	D	V	H	L	M	G	NST	NVS	-	NTT	NLS	NCS	NGT	NGS
NT7111	PSR SSR↓GL	N	N	N	D	V	N	L	M	G	NST	NVS	-	NTT	NVS	NCS	NGT	NGS
YC0220	PSR SSR↓GL	N	N	N	D	V	N	L	M	G	NST	NVS	-	NTT	NVS	NCS	NGT	NGS
YC0222	PSR SSR↓GL	N	N	N	D	V	N	L	M	G	NST	NVS	-	NTT	NVS	NCS	NGT	NGS
YC0277	PSR SSR↓GL	N	N	N	D	V	N	L	M	G	NST	NVS	-	NTT	NVS	NCS	NGT	NGS
YC0218	PSR SSR↓GL	N	N	N	D	V	N	L	M	G	NST	NVS	-	NTT	NVS	NCS	NGT	NGS
A/Anhui-Tianjiaan/ 11086/2022 (H9N2)	PSRSSR↓GL	N	N	N	D	V	N	L	M	G	NST	NVS	-	NTT	NVS	NCS	NGT	NGS
A/Anhui-Lujiang/39/ 2018 (H9N2)	PSRSSR↓GL	D	N	N	D	T	N	L	M	G	NST	NVS	-	NTT	NVS	NCS	NGT	NGS
A/Chicken/Shanghai/ F/98	PARSSR↓GL	N	A	N	T	A	N	Q	Q	G	NST	NVS	NRT	NTT	NVS	-	NGT	NGS
A/Duck/Hong Kong/Y280/97	PARSSR↓GL	N	A	N	T	T	N	L	Q	G	NST	NVS	NRT	NTT	NVS	-	NGT	-

Notes: The downward arrow (↓) marks the protease cleavage position within the HA cleavage site motif.

**Table 4:** Analysis of key amino acid residues and potential glycosylation sites in the NA protein of isolates.

Strain	Neck Deletion	Sialic Acid-Binding Sites			Potential Glycosylation Sites							
	63–65	368–370	399–401	431–433	69	86	146	200	234	306	368	402
NT7155	√	NSS	DSD	PQE	NST	NWS	NGT	NAT	NGT	-	NSS	NWS
ZJ1144	√	NSS	DGD	PQE	NST	NWS	NGT	NAT	NGT	NMT	NSS	-
CZ3090	√	NSS	DSD	PQE	NST	-	NGT	NAT	NGT	-	NSS	NWS
CZ3039	√	NSS	DSD	PQE	NST	-	NGT	NAT	NGT	-	NSS	NWS
NT7350	√	NSS	DSD	PQE	NST	NWS	NGT	NAT	NGT	-	NSS	NWS
CZ4061	√	NSS	DSD	PQE	NST	NWS	NGT	NAT	NGT	-	NSS	NWS
CZ3053	√	NSS	DSD	PQE	NST	-	NGT	NAT	NGT	-	NSS	NWS
NT7111	√	NSS	DSD	PQE	NST	NWS	NGT	NAT	NGT	-	NSS	NWS
YC0220	√	NDS	DSV	PQE	NST	NWS	NGT	NAT	NGT	-	NDS	-
YC0222	√	NDS	DSV	PQE	NST	NWS	NGT	NAT	NGT	-	NDS	-
YC0277	√	NDS	DSV	PQE	NST	NWS	NGT	NAT	NGT	-	NDS	-
YC0218	√	NDS	DSV	PQE	NST	NWS	NGT	NAT	NGT	-	NDS	-
A/Anhui-Tianjiaan/ 11086/2022(H9N2)	√	NGS	DSV	PQE	NST	NWS	NGT	NAT	NGT	-	NGS	-
A/AnhuiLujiang/39/ 2018(H9N2)	√	NGS	DSD	PQE	NST	NWS	NGT	NAT	NGT	-	NGS	NGS
A/Duck/Hong Kong/Y280/97(H9N2)	×	KDS	DSD	PQE	NST	NWS	NRT	NAT	NGT	-	-	-

Notes: The symbol √ indicates the presence of deletion at positions 63–65 in the HA neck region; × indicates no deletion at this region.

**Table 5:** HA titers of 12 H9N2 subtype AIV isolates against erythrocytes from different animals.

Strain Name	1% Erythrocyte Type					
	Chicken	Turkey	Guinea Pig	Horse	Sheep	Rabbit
NT7155	64	256	256	-	-	-
ZJ1144	128	1024	512	-	-	-
CZ3090	2	32	16	-	-	-
CZ3039	2	64	64	-	-	-
NT7350	8	128	128	-	-	-
CZ4061	512	2048	1024	-	-	-
CZ3053	128	512	512	32	16	16
NT7111	16	64	32	-	-	-
YC0220	32	128	128	-	-	-
YC0222	32	128	64	-	-	-
YC0277	16	128	128	-	-	-
YC0218	16	64	128	-	-	-
A/Anhui-Lujiang/39/2018 (H9N2)	128	1024	1024	-	-	-
A/Anhui-Tianjiaan/11086/2022(H9N2)	128	512	512	-	-	-

Note: ‘-’ indicates no agglutination; the values in the table represent the haemagglutination titer of the virus.

## Data Availability

The data that support the findings of this study are available from the corresponding author upon reasonable request.
